# Genetic Analysis of Substrain Divergence in Non-Obese Diabetic (NOD) Mice

**DOI:** 10.1534/g3.115.017046

**Published:** 2015-03-03

**Authors:** Petr Simecek, Gary A. Churchill, Hyuna Yang, Lucy B. Rowe, Lieselotte Herberg, David V. Serreze, Edward H. Leiter

**Affiliations:** * The Jackson Laboratory, Bar Harbor, Maine 04609; † Exploratory Statistics, DSS, AbbVie, North Chicago, Illinois 60064; ‡ Diabetes Research Institute, Düsseldorf, Germany

**Keywords:** type 1 diabetes, NOD mice, substrain divergence, exome capture, *Icam2*

## Abstract

The non-obese diabetic (NOD) mouse is a polygenic model for type 1 diabetes that is characterized by insulitis, a leukocytic infiltration of the pancreatic islets. During ~35 years since the original inbred strain was developed in Japan, NOD substrains have been established at different laboratories around the world. Although environmental differences among NOD colonies capable of impacting diabetes incidence have been recognized, differences arising from genetic divergence have not been analyzed previously. We use both mouse diversity array and whole-exome capture sequencing platforms to identify genetic differences distinguishing five NOD substrains. We describe 64 single-nucleotide polymorphisms, and two short indels that differ in coding regions of the five NOD substrains. A 100-kb deletion on Chromosome 3 distinguishes NOD/ShiLtJ and NOD/ShiLtDvs from three other substrains, whereas a 111-kb deletion in the *Icam2* gene on Chromosome 11 is unique to the NOD/ShiLtDvs genome. The extent of genetic divergence for NOD substrains is compared with similar studies for C57BL6 and BALB/c substrains. As mutations are fixed to homozygosity by continued inbreeding, significant differences in substrain phenotypes are to be expected. These results emphasize the importance of using embryo freezing methods to minimize genetic drift within substrains and of applying appropriate genetic nomenclature to permit substrain recognition when one is used.

The non-obese diabetic (NOD) mouse represents a premier animal model for the study of spontaneous insulitis and autoimmune type 1 diabetes. This inbred strain, first reported in 1980, was developed in Japan by selective breeding of outbred ICR:Jcl mice at the Shionogi Research Laboratories (Makino *et al.* 1980). The Central Laboratory for Experimental Animals (Japan) began receiving breeding stock from the NOD/Shi source colony for international distribution by 1986. However, before that time, NOD/Shi breeding stock from various sources had been obtained in two locations in the United States, one in Germany, and one in Australia (reviewed in Leiter 1998). Accumulation of new mutations fixed to homozygosity by inbreeding can be expected to produce significant substrain divergence over time, and potentially, differences in substrain characteristics. When NOD mice are maintained by inbreeding for at least 10 generations separately from the source colony (NOD/Shi), they are designated as substrains and receive either the colony holder’s and/or institution’s symbol. Among the currently most studied NOD substrains are NOD/ShiJcl (Central Laboratory for Experimental Animals Japan, Inc., http://www.clea-japan.com/en/animals/animal_b/b_06.html), NOD/ShiLtJ (The Jackson Laboratory, http://jaxmice.jax.org/strain/001976.html) and the NOD/ShiLtDvs substrain derived from it, NOD/MrkTac (Taconic, http://www.taconic.com/wmspage.cfm?parm1=871), and NOD/BomTac (TaconicEurope@taconic.com).

Phenotypic differences between and within NOD substrains have been observed (De Riva *et al.* 2013; Leiter 1993; Takayama *et al.* 1993). Given the strong role that environmental factors and especially the microbiome play in promoting or suppressing the T cell−mediated destruction of pancreatic beta cells in NOD mice (Markle *et al.* 2013), the possibility that substrain genetic differences also may account for marked variations in diabetes incidences among different colonies of NOD mice has remained an open question. In this regard, NOD males show the greatest variation in diabetes penetrance when compared across colonies, with the microbiome recently demonstrated as a major contributory factor (Markle *et al.* 2013).

The advantages and pitfalls of genetic analysis of closely related strains have been recently demonstrated in the comparison of C57BL/6J *vs.* C57BL/6N (Kumar *et al.* 2013) and BALB/cJ *vs.* BALB/cByJ (Sittig *et al.* 2014). The limited number of polymorphisms between substrains enables their manual curation and increases a chance for identification of single coding polymorphism responsible for the variation of the phenotype. An additional advantage to studying genomic comparisons within NOD substrains is the availability of BAC libraries for two of them, NOD/MrkTac and NOD/ShiLtJ (Steward *et al.* 2010); https://www.sanger.ac.uk/resources/mouse/nod/.

Here, we report the results of a screen for genetic drift among selected NOD substrains using a customized, high-density genotyping chip array combined with whole-exome sequencing.

## Materials and Methods

### Substrains

High molecular weight genomic DNA prepared from NOD/ShiJcl kidney was kindly provided by Dr. K. Hamaguchi (Oita University, Japan). NOD/ShiLtJ and NOD/ShiLtDvs genomic DNA was prepared from spleens by the JAX DNA Resource and from tail snips of NOD/MrkTac (kindly provided by Dr. L. Wicker, Cambridge University, UK) and the NOD/BomTac substrains (kindly provided by Dr. H.-J. Partke, Diabetes Research Institute, Düsseldorf, Germany). It should be noted that the nomenclature for NOD/LtJ and NOD/LtDvs was changed in 2007 by addition of the source colony descriptor “Shi”; although the MrkTac and BomTac substrains share this common origin, their substrain nomenclatures have not yet been changed to reflect this. The Lt substrain has been bred by Dr. E. Leiter at The Jackson Laboratory since 1984 and sent to its distribution arm in 1992. The Dvs substrain was separated from the Lt substrain in 1992 at The Jackson Laboratory, where it is maintained by Dr. D. Serreze as a research colony. The progenitors of the MrkTac substrain were derived from mice from Dr. Y. Mullen’s research colony at UCLA via Dr. L. Wicker (Merck Research Laboratories). The BomTac substrain breeding stock was obtained from a research colony maintained by Dr. L. Herberg (Diabetes Research Institute, Düsseldorf) who received the progenitors from Japan in 1984.

### Exome capture and sequencing

One microgram of each substrain DNA was fragmented using Covaris E220 (Covaris, Woburn, MA) to a range of sizes centered on 300 bp. The five precapture sequencing libraries were prepared using the NEBNext DNA Library Prep Master Mix Set for Illumina (New England BioLabs, Ipswich, MA) including a bead-based selection for inserts with an average size of 300 bp. The resulting precapture libraries were hybridized to the Roche NimbleGen Mouse Exome capture probe set (Roche NimbleGen, Madison, WI) according to the manufacturer’s instructions. Individually indexed library samples from NOD/ShiLtJ, NOD/LtDvs, and NOD/MrkTac were pooled for exome capture with NOD/Bom and NOD/Shi in a separate capture pool. The two final captured libraries were amplified by 18 cycles of polymerase chain reaction (PCR) using Phusion High Fidelity PCR mix (NEB). The resulting sequencing libraries were quantified by quantitative PCR, combined, and sequenced on an Illumina HiSequation 2500 (Illumina, San Diego, CA).

Raw reads in FASTQ format were aligned to the mouse reference genome (GRCm38, mm10) using bwa 0.5.9 aligner. Single-nucleotide polymorphisms (SNPs), short insertions, and deletions (Indels) were called by the UnifiedGenotyper of Genome Analysis Toolkit 3.1-1 (Depristo *et al.* 2011), validated by SAMtools 0.1.19 (Li *et al.* 2009) with heterozygous calls filtered out, manually checked in IGV 2.1 viewer (Broad Institute) and annotated in Ensembl Variant Effect Predictor. The identified polymorphisms were tested for presence in insulin-dependent diabetes (*Idd*) genetic regions as listed in Table 6.2 of (Ridgway *et al.* 2008). Note that because of the restriction on gap extension in bwa and the use of UnifiedGenotyper, the chance of identifying larger indels (>5 bp) is limited in this analysis. Data are deposited at the Sequence Read Archive, accession SRP045183.

### Mouse diversity array

The high density mouse diversity array (MDA) comprises more than 623,000 SNP probes plus an additional 916,000 invariant genomic probes targeted to genetic deletions or duplications (Yang *et al.* 2009).

DNA was prepared from tissue samples as described previously, and genotyped using MDA as previously described (Yang *et al.* 2009). Raw data (CEL files) are available at ftp://ftp.jax.org/petrs/MDA/raw data, see Supporting Information, Table S1 for CEL identifications, as implemented in genotypeSnps function of R package MouseDivGeno (Didion *et al.* 2012), with heterozygous calls filtered out. For detection of copy number variation (CNV), we used simple CNV function of the same package. Only long CNVs covering a number of probes can be discovered by this method (Bengtsson *et al.* 2008; Wang *et al.* 2007).

### PCR validation of Chr. 3 deletion

To validate the substrain-limited deletion on proximal Chr. 3, two pairs of PCR primers were designed and the products were Sanger sequenced on an ABI 3730 sequencer at JAX core services. The first pair of primers D3Jmp20 5′-TGTGGTGGACATTTGGGATA-3′ (forward), 5′-AGGCACAGGCAGATCATTCT-3′ (reverse) designed on either side of the approximately 110 kb deletion, gave a 362 bp band from NOD/ShiLt and no band from NON/LtJ. The deletion PCR product was Sanger sequenced to confirm the exact breakpoints. The second pair D3Jmp21 5′-ATCACAGGGTGATCACAGCA-3′ (forward) and 5′-TGTGTTCTTTTCACCCACCA-3′ amplify a product from within the deletion, and gave no product from NOD/ShiLt (region deleted) and a 469-bp band from NON/LtJ.

## Results

### Substrain genome comparisons by exome sequencing and MDA analysis

**Figure 1:**
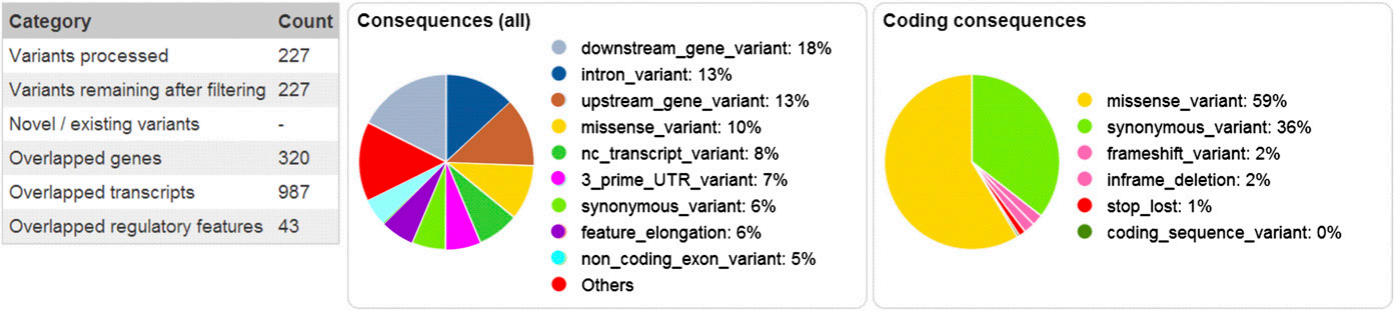
Consequences of polymorphisms identified by exome sequencing categorized by Ensembl Variant Effect Predictor. Totally, 172 SNPs + 55 indels = 227 variants are categorized and percentages of potential consequences is given (A) for all consequences (B) for consequences in coding region. See Ensembl Variant documentation for explanation of consequence categories (http://www.ensembl.org/info/genome/variation/predicted_data.html#consequences).

**Figure 2:**
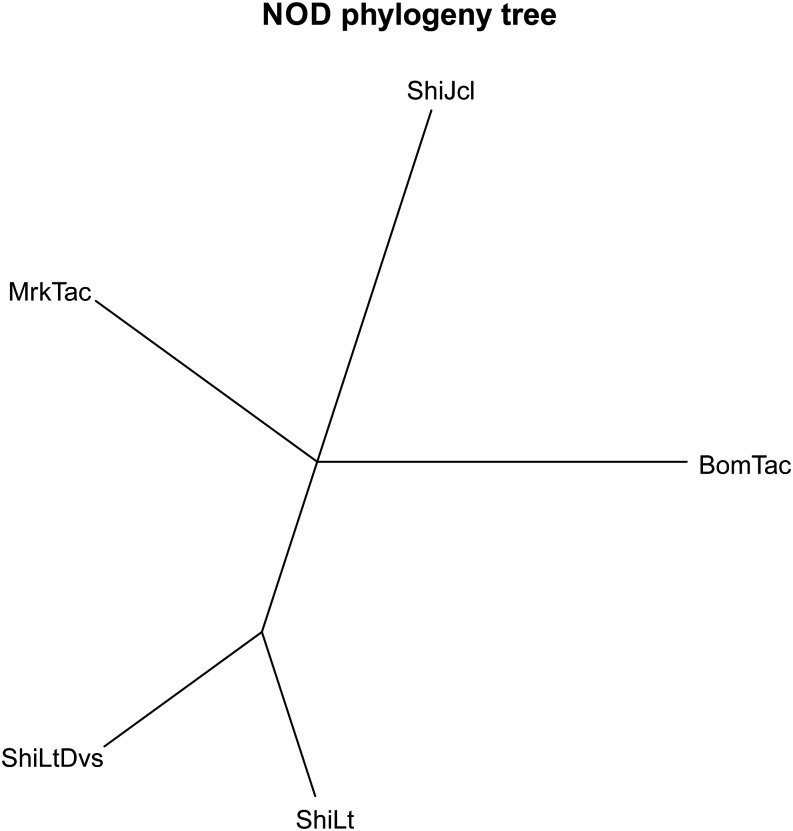
Phylogeny tree of NOD substrains. The length of edges in the tree corresponds to number of SNPs between substrains. NOD, non-obese diabetic; SNP, single-nucleotide polymorphism.

Table 1 shows a comparison of the numbers of SNPs and short indels distinguishing the exomes of the NOD/ShiLt, NOD/ShiLtDvs, NOD/MrkTac, NOD/BomTac, and NOD/ShiJcl substrains. The chromosomal locations are provided in Table S2 for SNPs and Table S3 for short indels. A summary of all polymorphisms and their consequences are shown in Figure 1. Substrain relationships based upon these polymorphisms were used to estimate a phylogeny tree under generalized time reversible model (Felsenstein 2004) (Figure 2). This analysis divides the substrains into two clusters; the NOD/ShiLtJ and NOD/ShiLtDvs that were separated most recently and the rest, NOD/ShiJcl, NOD/MrkTac, and NOD/BomTac. Test of the molecular-clock hypothesis, that length of edges is linearly dependent on time of separation, was rejected by a likelihood ratio test (*P* = 0.007). This could reflect differences in husbandry practices (colony size) resulting in different rates of fixation.

**Table 1:** Number of all SNPs, all indels, and coding SNPs + indels distinguishing each pair of substrains are compared with an approximate number of years since separation (exome sequencing only)

	All SNP					All Indels				
	BomTac	MrkTac	ShiJcl	ShiLtDvs	ShiLt	BomTac	MrkTac	ShiJcl	ShiLtDvs	ShiLt
BomTac	0	79	80	86	85	0	22	29	22	26
MrkTac	79	0	81	74	73	22	0	25	26	30
ShiJcl	80	81	0	89	88	29	25	0	31	27
ShiLtDvs	86	74	89	0	37	22	26	31	0	18
ShiLt	85	73	88	37	0	26	30	27	18	0

	Coding SNP + Indels					Years Since Separation (Approx.)				
	BomTac	MrkTac	ShiJcl	ShiLtDvs	ShiLt	BomTac	MrkTac	ShiJcl	ShiLtDvs	ShiLt
BomTac	0	33	33	25	33	0	28	27	27	27
MrkTac	33	0	30	22	30	28	0	28	28	28
ShiJcl	33	30	0	24	32	27	28	0	27	27
ShiLtDvs	25	22	24	0	14	27	28	27	0	19
ShiLt	33	30	32	14	0	27	28	27	19	0

SNP, single-nucleotide polymorphism.

**Figure 3:**
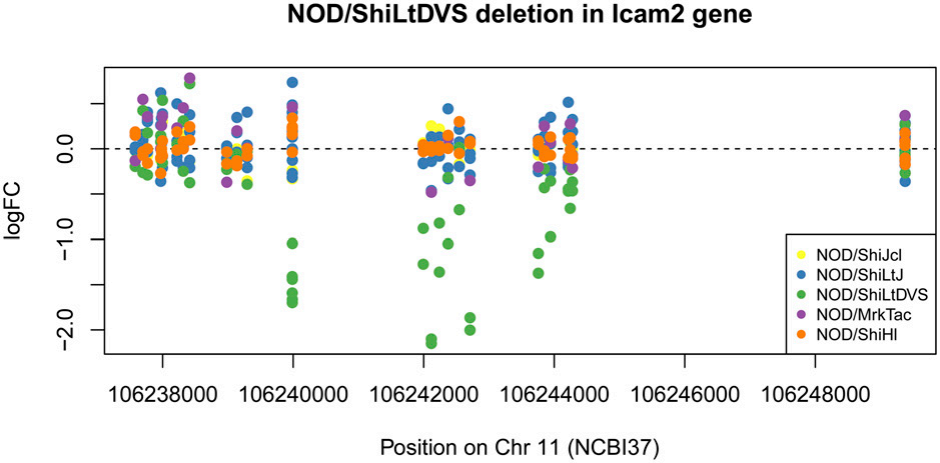
Scaled intensities of MDA probes along the *Icam2* region. Negative NOD/ShiLtDvs values suggest a deletion. See Figure S1. MDA, mouse diversity array; NOD, non-obese diabetic.

Not surprisingly given their more recent separation, the fewest polymorphisms distinguish LtDvs and LtJ. However, in the relatively short period since these two substrains separated, a deletion occurred in the Dvs *Icam2* (intracellular adhesion molecule2) gene on Chromosome (Chr.) 11 that now distinguishes this substrain from both the source LtJ stock and the other three substrains. The loss in intensity of MDA probes only in the Dvs *Icam2* region (Figure 3) was confirmed by exome sequencing as a deletion that included all exons except the last one (Figure S1). Flow cytometric analysis further confirmed the complete absence of ICAM-2 protein on Dvs substrain leukocytes. Although the *Icam1* gene is essential for diabetes development in NOD (Martin *et al.* 2001) the presence of this deletion shows that the *Icam2* gene in the Dvs substrain is dispensable for diabetogenesis. Flow cytometric analyses of congenic stocks built using NOD/ShiLtDvs as the recipient strain indicated those initiated before and after approximately the year 2000 are respectively ICAM-2 intact and deficient. This indicates the *Icam2* deletion presently characterizing the NOD/ShiLtDvs substrain occurred around the year 2000.

Compared with exome sequencing, MDA enables discovery of specific SNPs in noncoding regions and CNVs. In total, 48 SNPs and seven CNVs were detected, listed in Table S4 and Table S5. Among these, a 110,275-bp deletion on proximal Chr. 3 was unique to the LtJ and Dvs substrains. This deletion in a gene-poor area spanned 13 SNPs between 24,272,052 and 24,383,100 bases and was validated by Sanger sequencing across the deletion in NOD/ShiLt (see the section *Materials and Methods*). The only protein coding gene at this interval is *Naaladl2* (N-acetylated alpha-linked acidic dipeptidase-like 2), whose expression in mouse is primarily limited to the eye. No overt eye phenotype has been observed to differentiate these substrains. SNPs covering this deletion provide an excellent means for distinguishing the closely related LtJ and Dvs substrains from the other three more genetically divergent substrains.

Table S2 and Table S3 provide substrain distribution patterns from the whole-exome sequencing data for SNPs and indels, respectively. We identified 64 coding SNPs and two indels. Of those SNPs that effect a missense change in protein coding exons, many are associated with neuronal functions [for example, the CNS-restricted protein tyrosine phosphatase, receptor type 2 mutation (*Ptprt*, Chr. 2) limited to the LtJ substrain]. Some missense mutations are unique for a given substrain, *e.g.*, missense mutations in *Tmem163* and *Fam129b* (Chr. 1), *Tet2* (Chr. 3), *Cpz* and *Nsg1* (Chr. 5), *Prdm10* (Chr. 9), *Arsg* (Chr. 11), *Pcnx* and *Akr1c12* (Chr. 12), *Liph* (Chr. 16), and *Exo6* (Chr. 18) are limited to BomTac. The missense mutations are differentially distributed across the five substrains, so that it is not possible to predict whether any one of them alone affects a critical immunophenotype affecting diabetes penetrance. This was also the case for the substrain distribution of insertions and deletions summarized in Table S3. Whereas the locations and size of the indels were most closely matched in the closely-related LtJ and Dvs substrains (each with 9 deletions and 11 insertions), they were not all concordant across the genome. Comparison of the indels in the other three substrains indicated the independence of these genetic changes.

### PCR jumping

Sequencing reads from a given substrain at a specific SNP are expected to show close to 100% base call identity, consistent with the inbred nature of the strains. However, we also analyzed the frequency of rare allele calls, as a measure of sequence error rates or possible contamination between samples.

From the 172 SNP loci we selected 22 loci with high coverage [all five strains had at least 100 sequencing reads covering that base position (Table S6)]. There were no consistent sources of rare allele calls that would implicate sample or barcode contamination. Sequencing error rate was estimated as 0–0.33% (mean 0.05). However, for a given substrain, the presence of a minor variant (present as a major allele in a different substrain in the same exome capture pool) was observed at a significantly greater frequency, 0.7–3.8% (mean 1.6%). Every locus with more than 100 reads in all 5 strains showed this greater rate of rare allele calls within pools. This observation is consistent with previous reports of “Jumping PCR” ( Kircher *et al.* 2012; Ramos *et al.* 2012). This phenomenon occurs during postcapture PCR library amplification, wherein a nascent DNA strand switches templates from one allele to another. This “Jumping PCR” generates apparent rare allele calls that could be problematic in interpretation of sequence data from outbred animals or human populations, but for sequencing studies using inbred mice, this type of low-frequency artifact is readily distinguishable from the expected homozygous allele calls.

## Discussion

In the 34 years since the original report of the NOD/Shi strain in Japan, the genomes of the derivative colonies are clearly exhibiting genetic drift. From the results shown in Table 1, we can estimate that number of coding polymorphisms for a year of separation among NOD substrains is 0.74−1.22 (pair-wise comparisons among five substrains), or 0.19−0.31 for a generation (under assumption of four generations per year). Our results are similar to a recent paper (Simon *et al.* 2013) in which the authors compared C57BL/6J and C57BL/6N mouse strains separated around 220 generations ago that identified 34 SNPs and two indels in the coding region.

One of the major phenotypic differences distinguishing various colonies of NOD mice worldwide has been the penetrance of diabetes, particularly in males. With the exception of the low diabetes−incidence NOD/Wehi substrain, where a single recessive mutation may explain this now extinct substrain’s diabetes resistance (Baxter *et al.* 1993), the genetic basis for differential diabetes penetrance in the currently distributed NOD substrains, if any, is unknown. Although our study shows that each substrain carries unique indels or missense mutations that distinguish them from one another, no single one of these mutations has a known effect on diabetes penetrance or an immunophenotype related to it. The Chr. 3 deletion in a gene poor region in LtJ and Dvs is at least 1 Mb proximal to the *Idd3/Il2* locus known to be a major determinant of diabetes susceptibility in NOD mice (Yamanouchi *et al.* 2007). As noted in the section *Materials and Methods*, to avoid any ambiguity resulting from false calls, this analysis was exclusively focused on homozygous calls. Hence, this analysis has uncovered only a subset of all the genomic differences that must distinguish these five substrains as their genomes continue to drift over time (for example, recent heterozygous mutations not yet fixed to homozygosity).

In conclusion, we have documented genetic drift among NOD substrains that allows for distinguishing them genetically when necessary. The extent to which the polymorphisms identified potentially contribute to phenotypic differences *among substrains* remains unclear. This study only focused on variants fixed to homozygosity; additional heterozygous mutations likely are continuing to be fixed to homozygosity with successive generations of inbreeding. That such mutations may affect phenotype is clear. For example, a cohort of NOD/Jos mice received by other investigators in 1988 had diverged into high and low diabetes incidence sublines by 1993 (Takayama *et al.* 1993). Cryopreservation efforts to keep genetic drift to a minimum are clearly useful in maintaining a consistent phenotype (Taft *et al.* 2006). Finally, the existence of genetic divergence among NOD substrains emphasizes the importance of using the appropriate genetic nomenclature that permits identification of NOD colonies that have been separated from a source colony for 10 generations of inbreeding or more.

## References

[bib1] Baxter A. G. Hamilton F. Mandel T. E. Augustine C. Cooke A., 1993 Genetic basis for diabetes resistance in NOD/WEHI mice. Eur. J. Immunogenet. 20: 409–417. 9098409 10.1111/j.1744-313x.1993.tb00160.x

[bib2] Bengtsson H. Irizarry R. Carvalho B. Speed T. P., 2008 Estimation and assessment of raw copy numbers at the single locus level. Bioinformatics 24: 759–767. 18204055 10.1093/bioinformatics/btn016

[bib3] DePristo M. A. Banks E. Poplin R. Garimella K. V. Maguire J. R., 2011 A framework for variation discovery and genotyping using next-generation DNA sequencing data. Nat. Genet. 43: 491–498. 21478889 10.1038/ng.806 PMC3083463

[bib4] De Riva A. Varley M. C. Bluck L. J. Cooke A. Deery M. J., 2013 Accelerated turnover of MHC class II molecules in nonobese diabetic mice is developmentally and environmentally regulated in vivo and dispensable for autoimmunity. J. Immunol. 190: 5961–5971. 23677470 10.4049/jimmunol.1300551 PMC3785126

[bib5] Didion J. P. Yang H. Sheppard K. Fu C. P. McMillan L., 2012 Discovery of novel variants in genotyping arrays improves genotype retention and reduces ascertainment bias. BMC Genomics 13: 34. 22260749 10.1186/1471-2164-13-34 PMC3305361

[bib6] Felsenstein J., 2004 Inferring Phylogenies. Sinauer Associates, Sunderland, MA.

[bib7] Kircher M. Sawyer S. Meyer M., 2012 Double indexing overcomes inaccuracies in multiplex sequencing on the Illumina platform. Nucleic Acids Res. 40: e3. 22021376 10.1093/nar/gkr771 PMC3245947

[bib8] Kumar V. Kim K. Joseph C. Kourrich S. Yoo S. H., 2013 C57BL/6N mutation in cytoplasmic FMRP interacting protein 2 regulates cocaine response. Science 342: 1508–1512. 24357318 10.1126/science.1245503 PMC4500108

[bib9] Leiter E. H., 1993 The nonobese diabetic mouse: a model for analyzing the interplay between heredity and environment in development of autoimmune disease. Ilar News 35: 4–14.

[bib10] Leiter E. H., 1998 NOD mice and related strains: origins, husbandry, and biology, pp. 1–35 in *NOD Mice and Related Strains: Research Applications in Diabetes*, *AIDS*, *Cancer*, *and Other Diseases*, edited by Leiter E. H. Atkinson M. A. R.G. Landes, Austin.

[bib11] Li H. Handsaker B. Wysoker A. Fennell T. Ruan J., 2009 The Sequence Alignment/Map format and SAMtools. Bioinformatics 25: 2078–2079. 19505943 10.1093/bioinformatics/btp352 PMC2723002

[bib12] Makino S. Kunimoto K. Muraoka Y. Mizushima Y. Katagiri K., 1980 Breeding of a non-obese, diabetic strain of mice. Exp. Anim. 29: 1–8. 10.1538/expanim1978.29.1_1 6995140

[bib13] Markle J. G. Frank D. N. Mortin-Toth S. Robertson C. E. Feazel L. M., 2013 Sex differences in the gut microbiome drive hormone-dependent regulation of autoimmunity. Science 339: 1084–1088. 23328391 10.1126/science.1233521

[bib14] Martin S. van Den Engel N. K. Vinke A. Heidenthal E. Schulte B., 2001 Dominant role of intercellular adhesion molecule-1 in the pathogenesis of autoimmune diabetes in non-obese diabetic mice. J. Autoimmun. 17: 109–117. 11591119 10.1006/jaut.2001.0526

[bib15] Ramos E. Levinson B. T. Chasnoff S. Hughes A. Young A. L., 2012 Population-based rare variant detection via pooled exome or custom hybridization capture with or without individual indexing. BMC Genomics 13: 683. 23216810 10.1186/1471-2164-13-683 PMC3534616

[bib16] Ridgway W. M. Peterson L. B. Todd J. A. Rainbow D. B. Healy B., 2008 Gene-gene interactions in the NOD mouse model of type 1 diabetes. Adv. Immunol. 100: 151–175. 19111166 10.1016/S0065-2776(08)00806-7

[bib17] Simon M. M. Greenaway S. White J. K. Fuchs H. Gailus-Durner V., 2013 A comparative phenotypic and genomic analysis of C57BL/6J and C57BL/6N mouse strains. Genome Biol. 14: R82. 23902802 10.1186/gb-2013-14-7-r82 PMC4053787

[bib18] Sittig L.J. Jeong C. Tixier E. Davis J. Barrios-Camacho C.M., 2014 Phenotypic instability between the near isogenic substrains BALB/cJ and BALB/cByJ. Mamm. Genome 25: 564−572. 24997021 10.1007/s00335-014-9531-1 PMC4241159

[bib19] Steward C. A. Humphray S. Plumb B. Jones M. C. Quail M. A., 2010 Genome-wide end-sequenced BAC resources for the NOD/MrkTac() and NOD/ShiLtJ() mouse genomes. Genomics 95: 105–110. 19909804 10.1016/j.ygeno.2009.10.004 PMC2824108

[bib20] Taft R. A. Davisson M. Wiles M. V., 2006 Know thy mouse. Trends Genet. 22: 649–653. 17007958 10.1016/j.tig.2006.09.010

[bib21] Takayama Y. Ichikawa T. Maki T., 1993 Effect of STZ administration on islet isograft and allograft survival in NOD mice. Diabetes 42: 324–329. 8425668 10.2337/diab.42.2.324

[bib22] Wang K. Li M. Hadley D. Liu R. Glessner J., 2007 PennCNV: an integrated hidden Markov model designed for high-resolution copy number variation detection in whole-genome SNP genotyping data. Genome Res. 17: 1665–1674. 17921354 10.1101/gr.6861907 PMC2045149

[bib23] Yamanouchi J. Rainbow D. Serra P. Howlett S. Hunter K., 2007 Interleukin-2 gene variation impairs regulatory T cell function and causes autoimmunity. Nat. Genet. 39: 329–337. 17277778 10.1038/ng1958 PMC2886969

[bib24] Yang H. Ding Y. Hutchins L. N. Szatkiewicz J. Bell T. A., 2009 A customized and versatile high-density genotyping array for the mouse. Nat. Methods 6: 663–666. 19668205 10.1038/nmeth.1359 PMC2735580

